# A systematic review and meta-analysis of acupuncture in Parkinson's disease with dysphagia

**DOI:** 10.3389/fneur.2023.1099012

**Published:** 2023-05-26

**Authors:** Jing Wu, Yi Wang, Xueyan Wang, Yujia Xie, Weihong Li

**Affiliations:** ^1^Basic Medical School of Chengdu University of Traditional Chinese Medicine, Chengdu, China; ^2^Clinical Medical School of Chengdu University of Traditional Chinese Medicine, Chengdu, China

**Keywords:** acupuncture, Parkinson's disease, dysphagia, systematic review, meta-analysis

## Abstract

**Objective:**

The systematic review and meta-analysis aimed to comprehensively evaluate acupuncture's efficacy and safety in treating dysphagia in Parkinson's disease (PD).

**Methods:**

We searched PubMed, Cochrane Library, Embase, Web of Science, China Knowledge Infrastructure (CNKI), China Science Journal Database (VIP), Wan-fang Database, and the China Biomedical Literature Service System (CBM) for randomized controlled trials (RCTs) comparing the efficacy of acupuncture alone or in combination with control treatment in improving dysphagia by October 2022. The degree of dysphagia was the primary outcome indicator, with secondary outcomes including serum albumin (ALB) and hemoglobin (Hb) levels, the incidence of pneumonia, and adverse events. Two investigators independently extracted information according to the inclusion and exclusion criteria. Data synthesis was calculated by RevMan (V.5.4.1) software.

**Results:**

This study included ten randomized controlled trials with 724 patients. Most RCTs have a high or uncertain risk of bias due to the lack of a blinded design. Meta-analysis showed that acupuncture combined with control treatment was superior to control treatment alone in improving Videofluoroscopic Swallowing Study (VFSS) scores (MD: 1.48; 95% CI: 1.16, 1.81; *P* < 0.00001) and reducing Standardized Swallowing Assessment (SSA) scores (MD: −3.08; 95% CI: −4.01, −2.15; *P* < 0.00001). Acupuncture combined with control therapy has a more significant benefit in improving the clinical efficiency of dysphagia in PD (RR: 1.40; 95%CI: 1.25, 1.58; *P* < 0.00001). Compared to the control group without acupuncture, acupuncture improved the nutritional status of patients and increased their serum ALB (MD: 3.38, 95%CI: 1.83, 4.92, *P* < 0.00001) and Hb levels (MD: 7.66; 95%CI: 5.57, 9.75; *P* < 0.00001). Three RCTs reported that the rate of pulmonary infections in the acupuncture group was lower than without acupuncture intervention (RR: 0.29, 95% CI: 0.14, 0.63; *P* = 0.001).

**Conclusion:**

Acupuncture could be recommended as an adjunctive treatment for dysphagia in PD. However, due to the high risk of bias in the included studies, more high-quality evidence is needed to confirm the efficacy and safety of acupuncture for dysphagia in PD.

**Systematic review registration:**

https://www.crd.york.ac.uk/prospero/display_record.php?ID=CRD42022370221.

## 1. Introduction

Dysphagia is a highly associated non-motor symptom of Parkinson's disease (PD), attributed to autonomic and gastrointestinal dysfunction (1, 2). However, it is only in the last few years that the importance of dysphagia has been recognized and has become a hot topic of research (3). Dysphagia can appear at any point during Parkinson's disease (4, 5). The prevalence of dysphagia in PD ranges from 11 to 81%, depending on the disease stage, disease course, or assessment method (6). Swallowing disorders adversely affect the diet and medication intake of PD patients, making nutritional intake and medication efficacy not guaranteed, reducing patients' quality of life, and in severe cases, even pneumonia and asphyxia (7, 8). In particular, aspiration pneumonia due to swallowing disorders is one of the leading causes of death in all patients with PD syndrome (9, 10). In addition, patients with PD with dysphagia have a higher prevalence of affective symptoms such as fear and depression (11, 12). Therefore, treating dysphagia in patients with PD is of clinical importance.

The mechanism of dysphagia in PD is unclear and involves both dopaminergic and non-dopaminergic (13). Dopaminergic treatment is known to improve motor and pulmonary function in Parkinson's patients; however, the effect of dopamine on swallowing function remains controversial (14–18). As dysphagia often aggravates the progression of PD, compensatory and rehabilitative strategies are commonly used to maintain functional swallowing, minimize the incidence and mortality of malnutrition and pulmonary infection, and maintain a satisfactory quality of life (19, 20). The short-term effects of Compensatory strategies such as changing eating habits, adjusting swallowing posture, and swallowing training are significant, but the long-term consequences may not be immediate (21–24).

Acupuncture is a traditional treatment in China, characterized by simple operation and easy acceptance by patients. The efficacy of acupuncture has been clinically verified, widely used in treating PD worldwide (25, 26), and included in the expert consensus on dysphagia treatment in China (27). It has been confirmed by clinical research and systematic review that acupuncture treatment has a good effect in improving the symptoms of Parkinson's disease patients with dysphagia, reducing adverse reactions of drugs, and improving the quality of life of patients, and has attracted more and more attention (28, 29). However, the effectiveness of acupuncture for treating dysphagia in patients with PD has not been fully confirmed due to the lack of highly credible evidence. Therefore, we designed this meta-analysis to review and evaluate the effects of acupuncture on swallowing function in patients with PD, aiming to provide a reference for clinical treatment.

## 2. Methods

This systematic review was developed based on the Preferred Reporting Items for Systematic reviews and Meta-Analyses (PRISMA) and checked by the PRISMA checklist (Appendix 1). The method used in this systematic review has been previously registered in PROSPERO (CRD42022370221), which is available from https://www.crd.york.ac.uk/prospero/.

### 2.1. Data sources and search strategy

From the establishment of the database to October 2022, we searched four English databases (PubMed, Cochrane Library, Embase, Web of Science) and four Chinese databases [China Knowledge Infrastructure (CNKI), China Science Journal Database (VIP), Wan-fang Database, and China Biomedical Literature Service System (CBM)]. No restrictions on countries or types of articles. The search terms included Parkinson's disease, Parkinson's disorders, deglutition disorders, dysphagia, and acupuncture, and the specific search strategy is shown in Appendix 2. In addition, we manually searched references cited in the included studies, previously published systematic reviews, and others to make sure that no literature was missed.

### 2.2. Inclusion and exclusion criteria

According to the PICOS principles, the inclusion criteria for this study were as follows: (1) Participants: Patients with a definite diagnosis of Parkinson's disease and tested for swallowing function, with dysphagia as a clinical manifestation of difficulty eating or choking on water. The diagnostic criteria for PD refer to the Chinese Guidelines for Diagnosis and Treatment of Parkinson's Disease (2016 Revision) and the diagnostic criteria for PD formulated by the Movement Disorder Society in 2015 (30, 31). There are no restrictions on age, gender, course of the disease, race, etc. (2) Interventions: The experimental group received acupuncture as a stand-alone or adjunctive treatment. All methods of treating conditions by stabbing needles into patients according to acupoints and using acupuncture techniques are considered acupuncture therapy, including general acupuncture, electroacupuncture, warming acupuncture, thumbtack needle, neck needling, prick bleeding, etc. There is no restriction on the specific intervention time, acupuncture point, and treatment course. (3) Control: The control group may use conventional therapy, swallowing rehabilitation, sham acupuncture, neuromuscular electrical stimulation, and so on. (4) Outcomes: The degree of dysphagia is the primary outcome indicator. Swallowing function can be assessed by the videofluoroscopic swallowing study (VFSS) (the higher the score, the better the swallowing function), the standardized swallowing assessment scale (SSA) (the lower the score, the better the swallowing function), and the water swallow test. Secondary outcomes included serum albumin (ALB) and hemoglobin (Hb) levels, the incidence of pneumonia, and adverse events (AE). (5) Study type: Only randomized controlled trials (RCTs) were included.

Exclusion criteria were: (1) Previous dysphagia caused by stroke, malignant disease of the posterior pharynx, digestive tract diseases, etc. (2) Studies with unclear diagnostic or assessment criteria. (3) Acupuncture is combined with other Chinese medical methods (e.g., herbal medicine, tui na, acupressure, and others) to treat dysphagia. (4) The control group used Chinese medicine. (5) Duplicate published studies or studies with incomplete data that remain unavailable after contacting the original author.

### 2.3. Data screening and extraction

All included studies were imported into Endnote 20. Two professionally trained reviewers (Jing Wu and Yi Wang) examined all studies separately, excluding duplicate articles and those that did not meet the inclusion criteria and finally identifying studies that met the intended inclusion criteria. After extracting data on authors, year of publication, age, sample size, duration of disease, intervention method, acupuncture points, outcome indicators, and adverse effects, the two reviewers cross-checked to ensure the accuracy of the data. Any disagreements during the screening and data extraction process could be resolved with the assistance of a third assessor (Yu-jia Xie). For literature lacking information, the original authors were contacted for additions.

### 2.4. Risk of bias

Two reviewers (Jing Wu and Yi Wang) independently performed the risk of bias assessments according to the Cochrane Handbook for Systematic Reviews of Interventions (32). The evaluation consisted of 7 entries: random sequence generation, allocation concealment, blinding of participants and personnel, blinding of outcome assessors, incomplete outcome data, selective reporting, and other sources of bias. Each entry was assessed by the assessors and classified as “low risk,” “high risk,” or “uncertain.”

### 2.5. Data analysis

RevMan 5.4.1 provided by Cochrane Collaboration was used for data analysis. Relative risk (RR) was chosen as the statistic for dichotomous data, and mean difference (MD) as the effect indicator for continuous variables to obtain *P*-values and 95% confidence intervals (CI). We defined *P* < 0.05 as a statistically significant difference. When the heterogeneity test is performed, the *I*^2^ test is executed first and combined with quantified by the *I*^2^ statistic for evaluation. If the heterogeneity test result is *I*^2^ < 50%, there is no significant heterogeneity among the results, and the fixed effect model is used for data analysis. If *I*^2^ ≥ 50%, there is statistical heterogeneity among the results. After excluding apparent clinical and methodological heterogeneity, the random effect model was used for meta-analysis.

### 2.6. Subgroup analysis and sensitivity analysis

We considered that different types of acupuncture may have influenced the effectiveness of acupuncture, so we performed a subgroup analysis of the efficiency of varying needle types for treating dysphagia in PD. *P*-values ≥ 0.05 for the interaction indicated that the treatment effect did not differ significantly between subgroups. Sensitivity analysis was conducted when necessary.

## 3. Results

### 3.1. Literature selection

**Figure 1:**
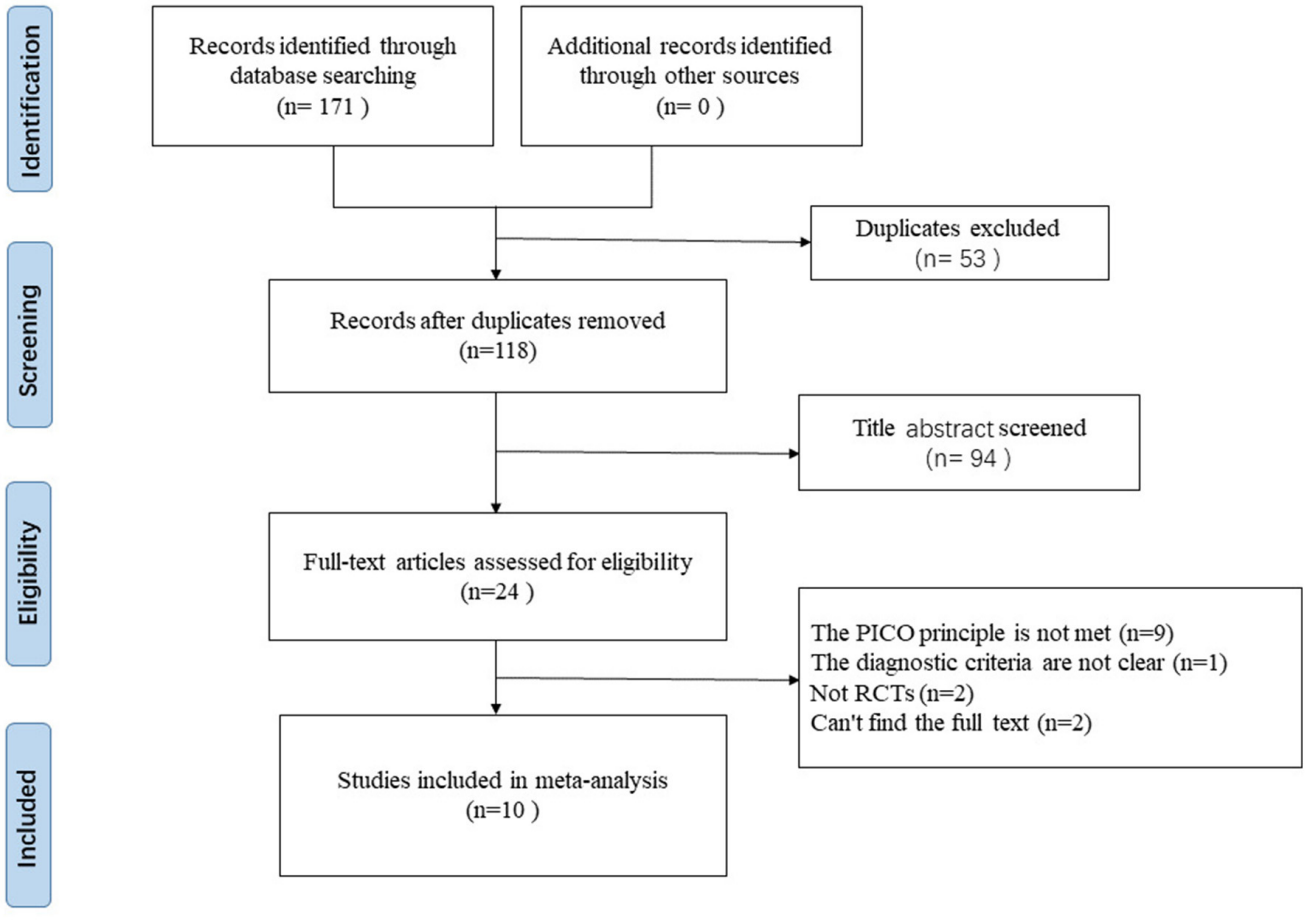
Flow diagram of searching and articles selection.

We searched a total of 171 papers from electronic databases. After excluding duplicate studies, 117 relevant studies were screened out. After reading the titles and abstracts of these studies, 24 relevant studies were identified. We read the full text before two independent reviewers performed further eligibility screening based on inclusion and exclusion criteria. The final 10 studies were included in the meta-analysis. The detailed literature screening process is shown in Figure 1.

### 3.2. Characteristics of the included literature

The 10 articles included in this study were all single-center randomized controlled trials conducted in China with 724 patients (362 in the experimental group and 362 in the control group) (29, 33–41). Two included literatures were master's theses (35, 41), and the remaining eight were journaled articles. The participants' ages ranged from 36 to 80 years. The average duration of dysphagia in PD was more than 1 year in seven of the 10 studies (29, 34, 35, 38–41), < 1 year in two studies (36, 37), and no duration was mentioned in the remaining studies. Among all included studies, eight studies compared acupuncture plus conventional management (CM) with CM alone (including Western medicine, swallowing training, and oral sensorimotor training) (29, 33–39), one study compared acupuncture plus CM with sham acupuncture plus CM (40), and one study compared acupuncture plus Western medicine with neuromuscular electrical stimulation (NMES) plus Western medicine (41). The videofluoroscopic swallowing study (VFSS) is the “gold standard” for measuring the function of swallowing (10). Five studies used the VFSS to evaluate patients' swallowing function (29, 34, 38, 39, 41). In these five studies, one study recorded the time parameters of the patient's intake of paste and liquids (29), one study recorded the time parameters of the patient's input of paste (41), and three studies recorded the total VFSS score using the penetration/aspiration scale (34, 38, 39). In addition, three studies used the standardized swallowing assessment scale (SSA) (39–41). Eight studies performed the water swallow test (29, 33–38, 40, 41), but one study had a different type of data than the others (38); one study was evaluated on various standards (34). For secondary outcomes, three studies (29, 34, 35) measured serum ALB and Hb levels reflecting nutritional status, three studies (35, 36, 41) documented the incidence of pulmonary infections after treatment, and one study (41) reported the safety of acupuncture. The specific characteristics of the included studies are shown in Table 1.

**Table 1:** Characteristics of included studies.

**References**	**Sample size (T/C)**	**Age (mean ± SD)**	**Disease duration**	**Invention**	**Duration of treatment**	**Acupoints**	**Control**	**Outcome**
Zhao et al. (33)	30/28	C: 36–70 T: 40–69	N/A	WA + FT	30 min each day	YaMen (DU15), LianQuan (RN23), JuQuan (EX-HN10), FengChi (GB20), RenZhong (DU26), NeiGuan (PC6), ZuSanLi (ST36)	FT	
Li et al. (34)	43/43	C: 58.46 ± 4.3 T: 59.37 ± 4.89	C: (6.01 ± 1.25) y T: (5.80 ± 1.43) y	EA + FT	5 times a week for 30 min, 4 weeks	LianQuan (RN23), YiFeng (SJ17), FengChi (GB20), WanGu (GB12), WaiYuYe, WaiJinJin	FT	
Miu (35)	28/28	C: 67.88 ± 8.53 T: 67.50 ± 9.70	C: (5.23 ± 2.14) y T: (5.25 ± 2.67) y	A + P + FT	Once a day, 4 weeeks.	LianQuan (RN23), ShangLianquan, JinJin YuYe (EX-HN12)	FT	
Shi (36)	56/56	C: 55.52 ± 1.14 T: 65.58 ± 1.16	C: (4.52 ± 0.26) w T: (4.54 ± 0.24) w	WA + FT	30 min each day	RenZhong (DU26), YaMen (DU15), LianQuan (RN23), NeiGuan (PC6), FengChi (GB20)	FT	
Wang et al. (37)	20/20	C: 52–70 T: 50–72	C: (228 ± 136) d T: (234 ± 140) d	A + P + FT	30 min each day, 20–30 days	SheJian, JinJin YuYe (EX-HN12), YanHouBi, BaiHui (DU20), LianQuan (RN23), HeGu (LI4), Quchi (LI11), WaiGuan (SJ5), TaiChong (LR3), ZuSanLi (ST36), SanYinJiao (SP6); point selection by syndrome differentiation	FT	
Wang (38)	45/45	C: 59 ± 10 T: 59 ± 10	C: (5.26 ± 1.02) y T: (5.31 ± 1.08) y	A + P + FT	5 times a week for 30 min, 4 weeks	ShenTing (DU24), BaiHui (DU20), ShangLianQuan, YinTang (DU29), TianZhu (BL10), FengChi (GB20), WaiGuan (SJ5), JinJin YuYe (EX-HN12), ZhaoHai (KI6), LieQue (LU7), YanHouBi	FT	
Wu et al. (29)	28/28	C: 65 ± 7 T: 63 ± 10	C: (5.4 ± 3.2) y T: (5.2 ± 3.3) y	A + P + FT	5 times a week for 30 min, 6 weeks	LianQuan (RN23), ShangLianQuan, FengChi (GB20), WaiGuan (SJ5), FengFu (DU16), YaMen (DU15), NeDaYing, JinJin YuYe (EX-HN12), YanHouBi	FT	
Wang et al. (39)	60/60	C: 52.0 ± 11 T: 54.0 ± 9.2	C: (1–2) y T: (1–2) y	A + FT	6 times a week for 30 min, 4 weeks	FengChi (GB20), YiMing (EX-HN13), GongXue, TunYan, LianQuan (RN23), WaiYuYe, WaJjinJin,	FT	
Yin et al. (40)	30/30	C: 65 ± 5.25 T: 63.17 ± 5.02	C: (4.85 ± 5.40) y T: (4.60 ± 5.65) y	TN + FT	Once every 2 days for 24 h each time	LianQuan (RN23), YiFeng (SJ17), JiaLianQuan, JiaJiXue (C3, C4, C5)	FT + SM	
Xie (41)	22/24	C: 64.8 ± 5.5 T: 65.3 ± 5.4	C: (6.26 ± 1.62) y T: (6.28 ± 1.50) y	A + M	6 times a week for 30 min, 4 weeeks	TaiXi (KI3), ZhaoHai (KI6), BaiHui (DU20), GuanYuan (RN4), SanYinJiao (SP6), TaiChong (LR3), HeGu (LI4), FengChi (GB20)XueHai (SP10), LianQuan (RN23), PangLianQuan	NMES + M	

C, Control; T, Treatment; y, year; d, day; w, week; A, Acupuncture; WA, Warming acupuncture; TN, Thumbtack needle; P, Prick bleeding; FT, Functional training; SM, Sham acupuncture; M, Medicine; NMES, Neuromuscular electrical stimulation; N/A, Not applicable., Water swallow test (WST);, Videofluoroscopic Swallowing Study (VFSS) scores;, Standardized Swallowing Assessment (SSA) scores;, Albumin (ALB);, Hemoglobin (Hb);, Incidence of pulmonary infection;, adverse events.

### 3.3. Acupuncture protocols included in the literature

**Figure 2:**
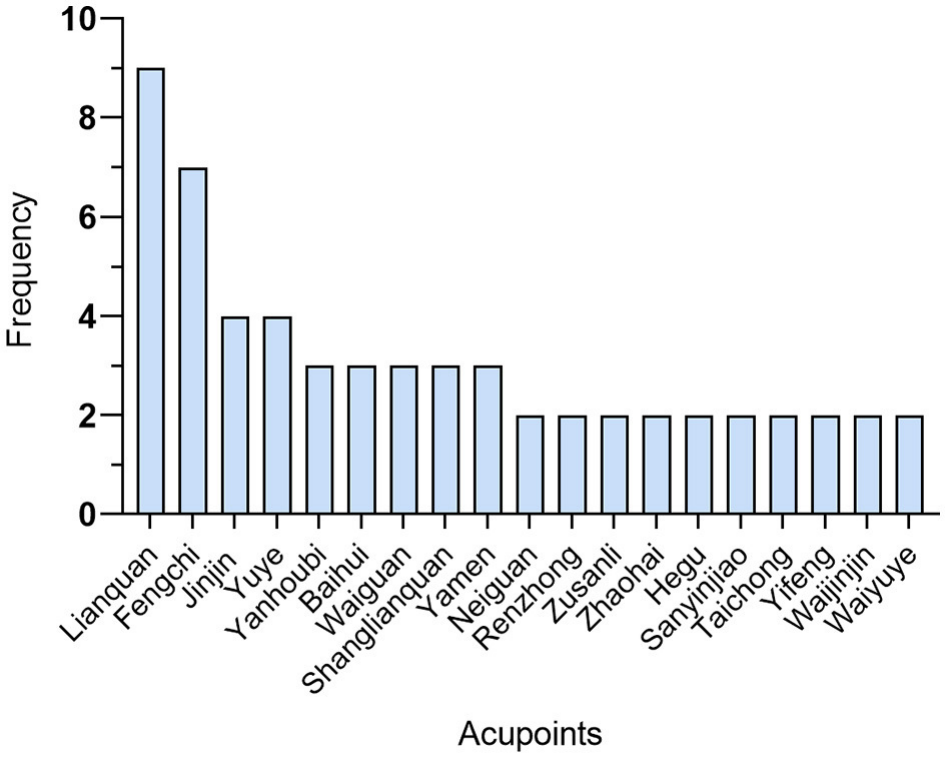
Frequency of commonly used acupoints.

Among the 10 included studies, warming acupuncture was used in two studies (33, 36), electroacupuncture was used in one study (34), manual acupuncture was used in two studies (38, 40), thumbtack needle was used in one study (40), and manual acupuncture combined with prick bleeding was used in four studies (29, 35, 37, 38). All the included literature described the selection of acupoints, as shown in Figure 2. Commonly used acupoints include Lianquan, Fengchi, Yamen, Baihui, Wangu, Jinjin, and Yuye. The needle retention time of body acupuncture is 30 min, and that of the intradermal needle is 24 h. Jinjin, Yuye, and Yanhoubi were punctured for bleeding without needle retention. The treatment frequency of acupuncture was once a day or every other day. The treatment period ranged from 20 days to 6 weeks. In all the studies, only two studies (37, 41) were treated based on syndrome differentiation, and the remaining studies applied fixed-point protocols. Of the included studies, only one study (35) provided information about acupuncturist certification, and eight studies (33–38, 40, 41) emphasized the sensation of De qi.

### 3.4. Risk of bias in the included literature

**Figure 3:**
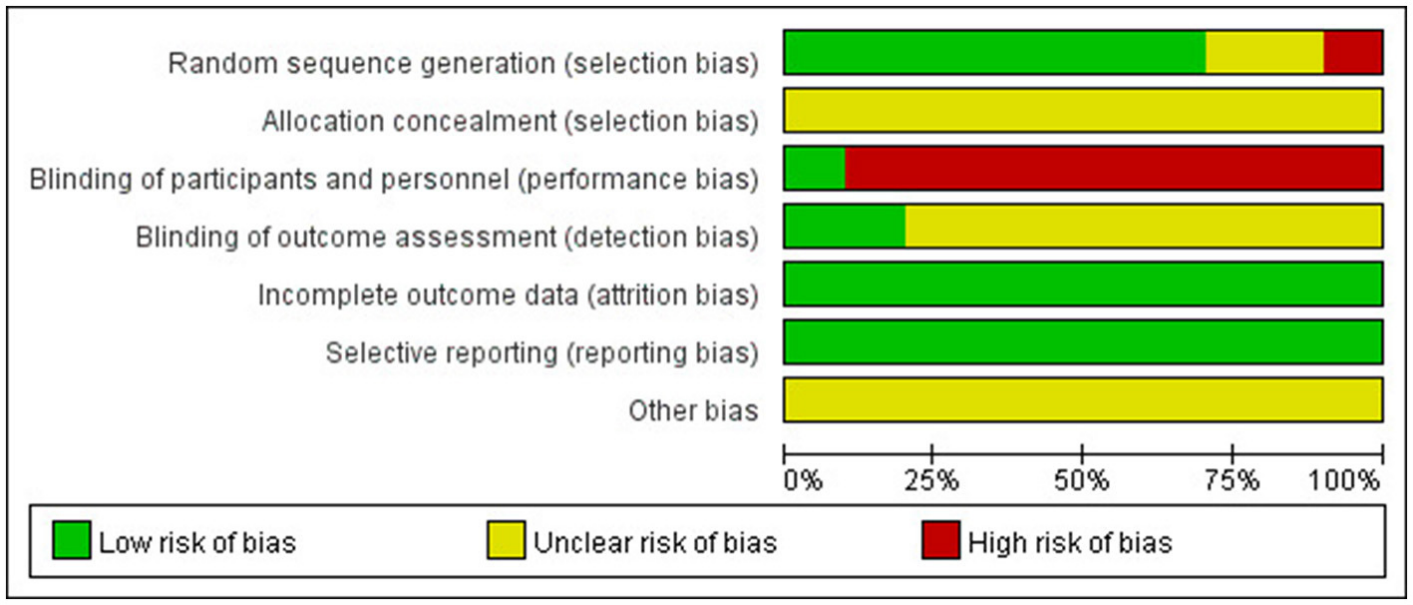
Risk of bias graph.

**Figure 4:**
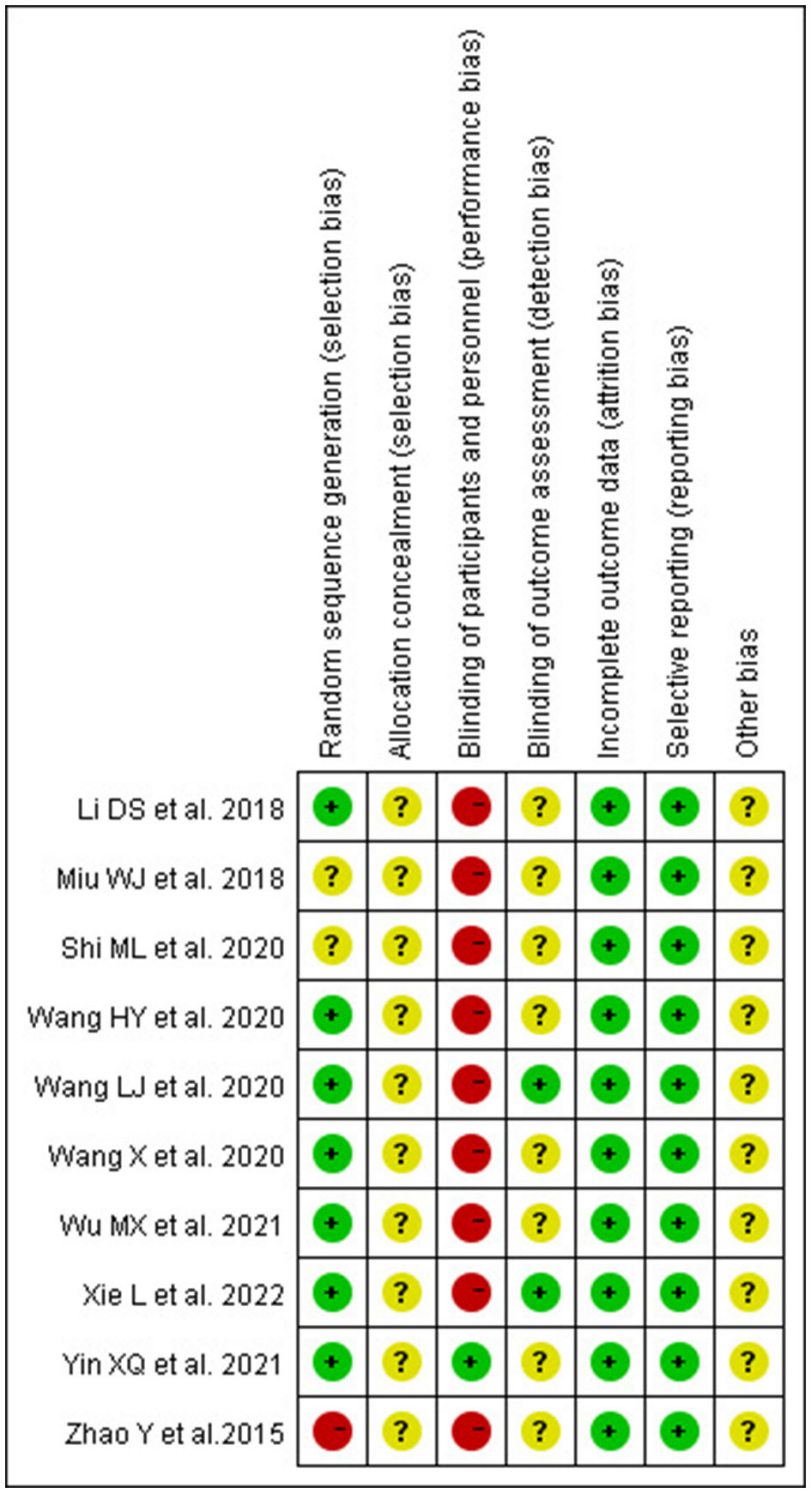
Risk of bias summary.

We assessed the risk of bias for all included articles. Two studies (35, 36) did not report a specific method of randomization, one study (33) used a randomization method with a high risk of bias according to visit order, and seven studies (29, 34, 37–41) reported the use of a random number table. None of the studies described allocation concealment and were judged to have an unclear risk of bias. Due to the specificity of acupuncture, only one study (40) told blinding patients to the use of sham acupuncture; the other studies did not mention the blinded design, which should be considered a high risk of bias. Two studies (39, 41) were blinded to the outcome indicator measure. All included RCTS had a low risk of bias in data completeness and selective reporting. The risk of bias assessment is summarized in Figures 3, 4.

### 3.5. Results of the meta-analysis

#### 3.5.1. VFSS scores

**Figure 5:**
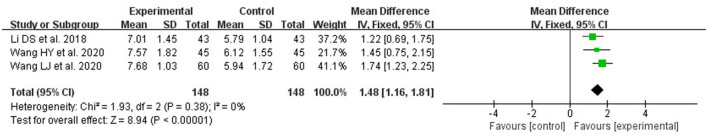
Forest plot of VFSS scores comparison between acupuncture and control group.

Five studies reported post-treatment VFSS scores (29, 34, 38, 39, 41), but we included only three studies due to differences in measurement methods across research (34, 38, 39). There was no significant heterogeneity between the three RCTs (*P* = 0.38, *I*^2^ = 0%), and the fixed-effects model showed a meaningful difference in VFSS scores between acupuncture combined with rehabilitation training (RT) and RT alone (MD: 1.48; 95% CI: 1.16, 1.81; *P* < 0.00001), indicating that acupuncture assisted treatment can significantly improve dysphagia in patients with PD, as shown in Figure 5.

#### 3.5.2. SSA scores

**Figure 6:**
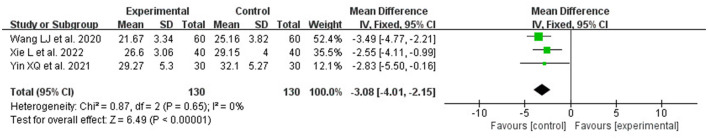
Forest plot of SSA scores comparison between acupuncture and control group.

SSA scores were reported in three studies (39–41). Since included studies showed no considerable heterogeneity (*P* = 0.65, *I*^2^ = 0%), fixed-effects models were used for analysis. The results showed that swallowing function was better in the acupuncture-treated group compared with the control group without acupuncture (MD: −3.08; 95% CI: −4.01, −2.15; *P* < 0.00001). See Figure 6.

#### 3.5.3. The efficiency of the water swallow test

**Figure 7:**
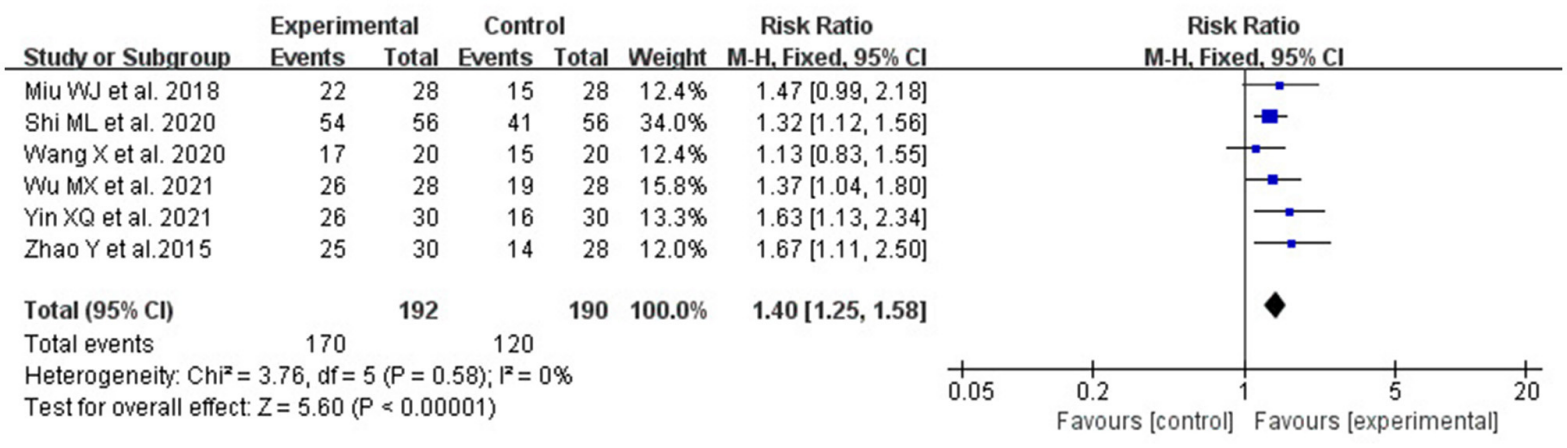
The forest plot shows a comparison of total efficiency rates between the acupuncture and the control group.

According to the grading and quantization standards determined in the Water swallow test (WST), the improvement of swallowing function was divided into four grades in six studies (29, 33, 35–37, 40), namely, cure: after treatment, the patient's swallowing disorder completely disappeared, the WST results rose to Grade I (swallow the water smoothly in one go), and there were no other discomfort symptoms; Remarkable effect: after treatment, the patient's dysphagia disappeared, and the WST results rose to Grade I–II (swallow without choking in two or more times, without other discomfort symptoms); Effective: After treatment, the patient's dysphagia has been improved, and the WST is Grade II–III (swallow in one go but with choking), with slight discomfort; Ineffective: The swallowing disorder did not improve or even worsen after treatment, and the WST result was higher than Grade III (choked frequently or could not swallow it all). The total effective rate is the sum of the number of cured, remarkable effects, and influential people as a percentage of the total number of people. As there was no significant heterogeneity between these studies, a meta-analysis was performed using a fixed-effects model (*P* = 0.58, *I*^2^ = 0%). The results showed that patients who received acupuncture combined with RT had more improvement in swallowing function compared with RT alone (RR: 1.40; 95% CI: 1.25, 1.58; *P* < 0.00001; Figure 7).

**Figure 8:**
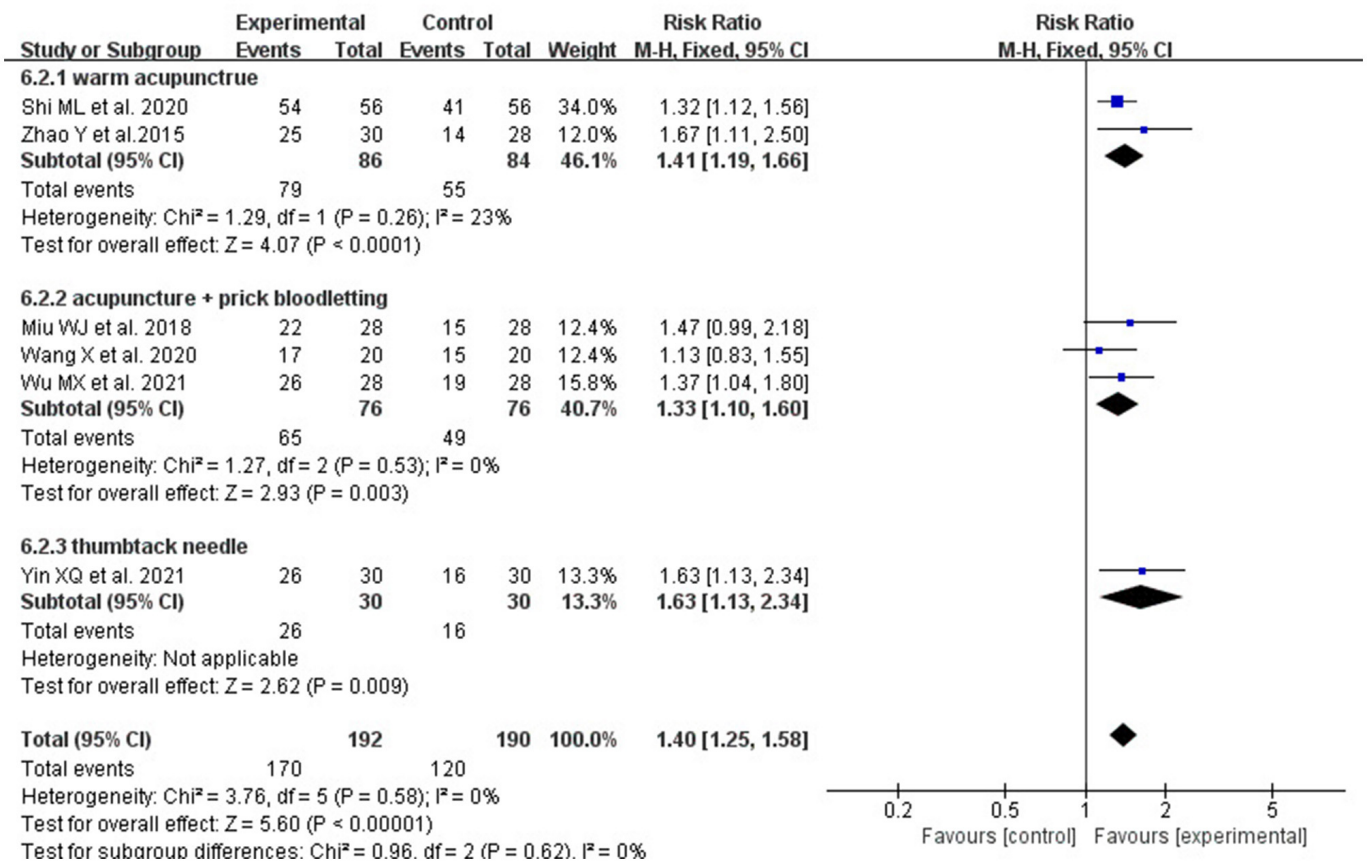
The forest plot shows a comparison of effectiveness in treating Parkinson's dysphagia between the acupuncture and the control group, based on a subgroup analysis of different acupuncture methods.

We divided the included studies into two subgroups according to the type of acupuncture to discuss the efficacy of acupuncture due to the variety of acupuncture methods in the experimental group. As shown in Figure 8, compared with RT alone, warming acupuncture plus RT (2 studies, RR: 1.41; 95% CI: 1.19, 1.66; *P* < 0.0001; heterogeneity: *I*^2^ = 23%, *P* = 0.26), prick bleeding plus rehabilitation (3 studies, RR: 1.33; 95% CI: 1.10, 1.60; *P* = 0.003; heterogeneity: *I*^2^ = 0%, *P* = 0.53) and thumbtack needle plus RT (1 study, RR: 1.63; 95% CI: 1.13, 2.34; *P* = 0.009;) both significantly enhanced the effective rate.

#### 3.5.4. Nutritional status

**Figure 9:**
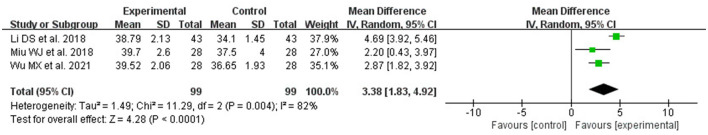
Forest plot of ALB level in comparison between acupuncture group and control.

**Figure 10:**
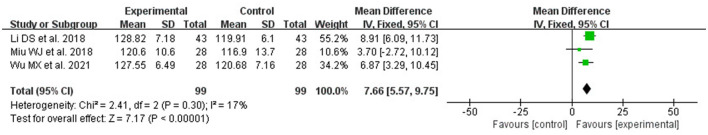
Forest plot of Hb level in comparison between acupuncture group and control.

The three included studies (29, 34, 35) assessed the nutritional status of the treatment and control groups through patient serum ALB and Hb levels. The statistical data showed that acupuncture combined with RT had a remarkable effect on ALB level (MD: 3.38, 95%CI:1.83, 4.92, *P* < 0.00001; heterogeneity: *I*^2^ = 82%, *P* = 0.004; Figure 9) and Hb level (MD: 7.66; 95% CI: 5.57, 9.75; *P* < 0.00001;f heterogeneity: *I*^2^ = 17%, *P* = 0.30; Figure 10) under conventional drug treatment, which indicated that acupuncture could improve the nutritional status of patients with dysphagia in PD.

#### 3.5.5. Incidence of pulmonary infections

**Figure 11:**
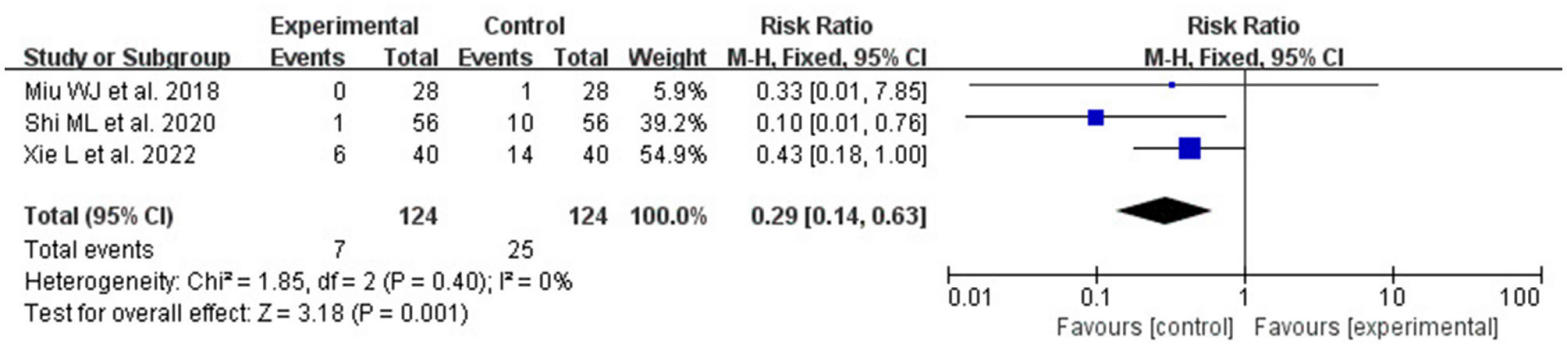
Forest plot comparing the incidence of pulmonary infection between the acupuncture and the control group.

The incidence of pulmonary infection is reported in three articles (35, 36, 41). The results of the fixed-effect model analysis showed that the incidence of pneumonia in the acupuncture group was significantly lower than that in the non-acupuncture group (RR: 0.29, 95% CI: 0.14, 0.63, *P* = 0.001; heterogeneity: *I*^2^ = 0%, *P* = 0.40; Figure 11).

#### 3.5.6. Adverse events

Of the ten included studies, only one reported no related acupuncture adverse events during the trial (41), and the remaining nine studies did not report adverse events.

### 3.6. Publication bias

Funnel plots were not used to investigate publication bias because of the limited number of included studies (< 10 trials).

## 4. Discussion

### 4.1. Main results and analysis

This systematic review included 10 RCTs that evaluated the patient's swallowing function, nutritional status, and incidence of pneumonia. Our study showed that acupuncture combined with rehabilitation training increased the effectiveness of improving swallowing function in patients with swallowing disorders in Parkinson's disease and could lead to significant improvement in the patient's swallowing function and reduced the incidence of pulmonary infections. In addition, patients with dysphagia are often accompanied by malnutrition, and the nutritional level is closely related to the prognosis of patients. The study on the correlation between dysphagia and the nutritional status of PD patients shows that the better the nutritional status of patients, the better the prognosis (42). ALB and Hb have the characteristics of convenient and rapid detection in the clinic, and are widely used to evaluate nutritional status. In malnourished patients, low albumin levels have been long-standing (43, 44). According to the results of Meta-analysis, even in the presence of chronic inflammation, several blood biomarkers including albumin, prealbumin and hemoglobin are useful biochemical indicators of adult malnutrition (45). When acupuncture is combined with the treatment measures of the control group, the serum ALB and Hb levels of patients are significantly improved. Thus, the results of this systematic review support acupuncture as an augmentation approach to improve dysphagia in Parkinson's disease. We did not obtain sufficient evidence regarding the effectiveness of acupuncture alone, which may be related to the synergistic effects of acupuncture with other therapies.

The Water Swallow Test and VFSS are commonly used clinical assessment methods of dysphagia, among which VFSS is recognized as the gold standard for the diagnosis of dysphagia (46). However, it needs special equipment, requires the subject to have a certain physical strength, can cooperate with the examination, and is radioactive in operation, thus posing the risk of aspiration of contrast agent, which limits its clinical application to a certain extent. The most important complication of dysphagia is risk for aspiration, so the detection of misophagia is the main purpose of clinical evaluation. A positive assessment of SSA score may provide a preliminary indication that a patient may have swallowed incorrectly, but since this is only a preliminary assessment and screening, it is necessary to refer the patient to an experienced language therapist for reassessment and further examination to identify dysphagia, which is one of the reasons why only a small number of studies use the SSA scale (47, 48).

### 4.2. Mechanism of acupuncture

Dysphagia is a serious adverse factor in the prognosis of PD patients and a significant cause of death (49). It has been suggested that various pathological changes involving nerves and muscles during the progression of PD patients can lead to impaired neuromodulation at any level of the peripheral nerves, brainstem swallowing centers, cerebral cortex, and subcortical centers, resulting in PD dysphagia (50, 51). Autopsy reports from PD patients show that α -synuclein is present in the peripheral sensory nerves of dysphagia patients and in the motor nerves that dominate the pharyngeal muscles, compared to patients without dysphagia (49, 52). There is a belief that although the central or peripheral nervous system in PD patients can reorganize structurally or functionally, this remodeling function does not occur naturally and needs to be achieved by receiving stimulation (29). Some studies have shown acupuncture can stimulate the supraglottic and parasympathetic nerves, increase cerebral blood flow in patients, promote the repair and reconstruction of pharyngeal reflex arc function, and thus enhance swallowing function. (53–58). Acupuncture also enhances the excitability of the central nervous system, coordinates the fine movements of the tongue and pharynx, improves the paralysis of the pharyngeal muscles, and further improves dysphagia (59–62). Qi Ling et al. showed that electroacupuncture could reduce the content of α -synuclein by inhibiting neuritis reaction, slowing down the apoptosis rate of dopaminergic neurons in the substantia nigra, improving the dopaminergic pathway, and thus promoting swallowing function (62).

### 4.3. Clinical effects

“Where the acupoints are located, the indications are located” is one of the roles of acupoints. The ability of an acupoint to treat diseases in its location and the adjacent organs, tissues, and organs is a common feature of all acupoints (63). This article showed that acupuncture has good effect on swallowing function in patients with dysphagia in PD. Acupuncture is mainly taken from the posterior pharynx and head, and the commonly used acupoints are Lianquan, Fengchi, Jinjin, and Yuyi. Puncture and bloodletting of the posterior pharyngeal wall is also commonly used. Follow-up studies should further investigate and screen stationary and effective acupuncture points to form a fixed localized acupuncture treatment plan to benefit more patients with dysphagia in PD. “Syndrome differentiation” is the basic principle of TCM (64). Wang et al. (37) and Xie (41) selected different acupuncture points for various symptoms of the patients, fully reflecting the personalized treatment of TCM and the treatment policy of seeking the fundamental cause of the disease.

Overall, this study is the first systematic evaluation and meta-analysis of the effectiveness and safety of acupuncture for Parkinson's swallowing disorder. We hope to provide doctors with a range of treatment strategies and help them design individualized interventions. According to the above results, doctors can develop the most appropriate approach for dysphagia in PD based on proximal acupoint selection and Syndrome differentiation, combined with the proper acupuncture method.

### 4.4. Limitations

Although this study followed the criteria stated in PRISMA, there are still some limitations. First, even though the meta-analysis shows no obvious heterogeneity, all the included studies are single-center RCTs in China, and the small sample size and the diversity of treatment methods may lead to certain potential biases, thus affecting the reliability of the results. Second, due to the specific nature of acupuncture therapy, studies are difficult to implement, blinding for participants and personnel, and there is a high risk of bias. Third, as only one of the included studies mentioned the absence of adverse effects and none of the other studies reported on the adverse effects of acupuncture, there was no systematic review of the possible acupuncture problems during treatment. Hence, to more comprehensively and objectively evaluate the efficacy of acupuncture in treating dysphagia in Parkinson's disease, future studies need to raise the sample size, provide reasonable allocation concealment and blinded design for trials, and provide more comprehensive reference information for subsequent research studies.

## 5. Conclusion

Acupuncture is effective as a complementary therapy for dysphagia in PD, not only improving patients' swallowing function but also enhancing their nutritional status and reducing the incidence of pneumonia. However, due to the high risk of bias in the included studies, the results should be interpreted with caution, and multicenter, more rigorous, and high-quality RCTs are necessary for subsequent analyses.

## Data availability statement

The original contributions presented in the study are included in the article/Supplementary material, further inquiries can be directed to the corresponding author.

## Author contributions

JW and XW selected a topic for the study. WL revised the manuscript. JW and YW conducted data extraction and quality assessment, completed the data synthesis, drafted the manuscript, and performed the search strategy. YX arbitrated in cases of disagreement and ensured the absence of errors. All authors have developed the search strategy, read, and approved the manuscript.
