# Supplementary material for: A systematic review and meta-analysis of acupuncture in Parkinson's disease with dysphagia
Source: Front Neurol. 2023 May 26;14:1099012. doi: 10.3389/fneur.2023.1099012 (PMC10251408; doi:10.3389/fneur.2023.1099012)
Supplement: Supplementary file 1 [file Data_Sheet_1.docx]

Supplementary Material

# Appendix 1: Search Strategy

**PubMed**

#1 Parkinson's disease[MeSH Terms]

#2 ((((((((Disease of Parkinson[Title/Abstract]) OR (Parkinson Disorders[Title/Abstract])) OR (Parkinson Disorders[Title/Abstract])) OR (PD[Title/Abstract])) (Parkinson's Disease[Title/Abstract])) OR (Idiopathic Parkinson* Disease[Title/Abstract])) OR (Lewy Body Parkinson* Disease[Title/Abstract])) OR (Primary Parkinsonism[Title/Abstract])) OR (Paralysis Agitans[Title/Abstract])

#3 #1 OR #2

#4 Deglutition Disorders[MeSH Terms]

#5 (((Deglutition Disorders[Title/Abstract]) OR (Dysphagia[Title/Abstract])) OR (Swallowing Disorder*[Title/Abstract])) OR (Swallowing difficulty[Title/Abstract])

#6 #4 OR #5

#7 Acupuncture[MeSH Terms]

#8 ((((((Acupuncture[Title/Abstract]) OR (moxibustion[Title/Abstract])) OR (Needle therapy[Title/Abstract])) OR (Needl*[Title/Abstract])) OR (Electroacupuncture[Title/Abstract])) OR (Zhen jiu[Title/Abstract])) OR (Zhen ci[Title/Abstract])

#9 #7 OR #8

#10 #3 AND #6 AND #9

**Embase**

#1 'parkinsons disease'/exp

#2 ('disease of parkinson':ab,ti OR 'parkinson disorders':ab,ti OR 'parkinsons disease':ab,ti OR 'idiopathic parkinson* disease':ab,ti OR 'PD':ab,ti OR 'lewy body parkinson* disease':ab,ti OR 'primary parkinsonism':ab,ti OR 'paralysis agitans':ab,ti)

#3 #1 OR #2

#4 'dysphagia'/exp

#5 ('deglutition disorders'/exp OR 'deglutition disorders':ab,ti OR dysphagia:ab,ti OR 'swallowing disorder*':ab,ti OR 'swallowing difficulty':ab,ti)

#6 #4 OR #5

#7 'acupuncture'/exp

#8 (‘acupuncture’:ab,ti OR ‘moxibustion’:ab,ti OR 'needle therapy':ab,ti OR needl*:ab,ti OR electroacupuncture:ab,ti OR 'zhen jiu':ab,ti OR 'zhen ci':ab,ti)

#9 #7 OR #8

#10 #3 AND #6 AND #9

**Cochrane Library**

#1 MeSH descriptor: [acupuncture] explode all trees

#2 (acupuncture OR moxibustion OR ‘needl* therapy' OR ‘needl*’ OR electroacupuncture OR 'zhen jiu' OR 'zhen ci') :ti,ab,kw

#3 #1 OR #2

#4 MeSH descriptor: [parkinsons disease] explode all trees

#5 ('disease of parkinson' OR 'parkinson disorders' OR 'parkinsons disease' OR 'idiopathic parkinson* disease' OR 'lewy body parkinson* disease' OR 'primary parkinsonism' OR 'paralysis agitans' OR 'PD' ) :ti,ab,kw

#6 #4 OR #5

#7 MeSH descriptor: [dysphagia] explode all trees

#8 MeSH descriptor: [deglutition disorders] explode all trees

#9 (‘Swallowing Disorder*’ OR ‘Swallowing difficulty’) :ti,ab,kw

#10 #7 OR #8 OR #9

#11 #3 AND #6 AND #10

**Web of Science**

((((((TS=(Parkinson's disease)) OR TS=(Disease of Parkinson)) OR TS=(Parkinson Disorders)) OR TS=(Idiopathic Parkinson* Disease)) OR TS=(Lewy Body Parkinson* Disease)) OR TS=(Primary Parkinsonism)) OR TS=(Paralysis Agitans) AND (((TS=(Deglutition Disorders)) OR TS=(Dysphagia)) OR TS=(Swallowing Disorder*)) OR TS=(Swallowing difficulty) AND ((((((TS=(Acupuncture)) OR TS=(moxibustion)) OR TS=(Needle therapy)) OR TS=(Needl*)) OR TS=(Electroacupuncture)) OR TS=(Zhen jiu)) OR TS=(Zhen ci)

**CNKI**

( ( ( ( (主题=帕金森病 或者 题名=帕金森病 或者 v_subject=中英文扩展(帕金森病) 或者 title=中英文扩展(帕金森病)) 或者 (主题=帕金森氏病 或者 题名=帕金森氏病 或者 v_subject=中英文扩展(帕金森氏病) 或者 title=中英文扩展(帕金森氏病)) ) 或者 ( (旧版主题=帕金森 或者 keyword=中英文扩展(帕金森) 或者 title=中英文扩展(帕金森) 或者 abstract=中英文扩展(帕金森)) 或者 (旧版主题=帕金森病 或者 keyword=中英文扩展(帕金森病) 或者 title=中英文扩展(帕金森病) 或者 abstract=中英文扩展(帕金森病)) ) ) 或者 ( (旧版主题=帕金森综合征 或者 keyword=中英文扩展(帕金森综合征) 或者 title=中英文扩展(帕金森综合征) 或者 abstract=中英文扩展(帕金森综合征)) 或者 (旧版主题=帕金森症 或者 keyword=中英文扩展(帕金森症) 或者 title=中英文扩展(帕金森症) 或者 abstract=中英文扩展(帕金森症)) ) ) 或者 ( (旧版主题=震颤麻痹 或者 keyword=中英文扩展(震颤麻痹) 或者 title=中英文扩展(震颤麻痹) 或者 abstract=中英文扩展(震颤麻痹)) 或者 (旧版主题=颤证 或者 keyword=中英文扩展(颤证) 或者 title=中英文扩展(颤证) 或者 abstract=中英文扩展(颤证)) ) ) 并且 ( ( ( (主题=吞咽障碍 或者 题名=吞咽障碍 或者 v_subject=中英文扩展(吞咽障碍) 或者 title=中英文扩展(吞咽障碍)) 或者 (主题=吞咽困难 或者 题名=吞咽困难 或者 v_subject=中英文扩展(吞咽困难) 或者 title=中英文扩展(吞咽困难)) ) 或者 ( (旧版主题=吞咽障碍 或者 keyword=中英文扩展(吞咽障碍) 或者 title=中英文扩展(吞咽障碍) 或者 abstract=中英文扩展(吞咽障碍)) 或者 (旧版主题=吞咽困难 或者 keyword=中英文扩展(吞咽困难) 或者 title=中英文扩展(吞咽困难) 或者 abstract=中英文扩展(吞咽困难)) ) ) 或者 ( (旧版主题=吞咽功能 或者 keyword=中英文扩展(吞咽功能) 或者 title=中英文扩展(吞咽功能) 或者 abstract=中英文扩展(吞咽功能)) 或者 (旧版主题=吞咽 或者 keyword=中英文扩展(吞咽) 或者 title=中英文扩展(吞咽) 或者 abstract=中英文扩展(吞咽)) ) ) 并且 ( ( ( ( ( ( ( (主题=针灸 或者 题名=针灸 或者 v_subject=中英文扩展(针灸) 或者 title=中英文扩展(针灸)) 或者 (主题=针刺 或者 题名=针刺 或者 v_subject=中英文扩展(针刺) 或者 title=中英文扩展(针刺)) ) 或者 ( (旧版主题=针灸 或者 keyword=中英文扩展(针灸) 或者 title=中英文扩展(针灸) 或者 abstract=中英文扩展(针灸)) 或者 (旧版主题=针刺 或者 keyword=中英文扩展(针刺) 或者 title=中英文扩展(针刺) 或者 abstract=中英文扩展(针刺)) ) ) 或者 ( (旧版主题=毫针 或者 keyword=中英文扩展(毫针) 或者 title=中英文扩展(毫针) 或者 abstract=中英文扩展(毫针)) 或者 (旧版主题=针法 或者 keyword=中英文扩展(针法) 或者 title=中英文扩展(针法) 或者 abstract=中英文扩展(针法)) ) ) 或者 ( (旧版主题=电针 或者 keyword=中英文扩展(电针) 或者 title=中英文扩展(电针) 或者 abstract=中英文扩展(电针)) 或者 (旧版主题=温针 或者 keyword=中英文扩展(温针) 或者 title=中英文扩展(温针) 或者 abstract=中英文扩展(温针)) ) ) 或者 ( (旧版主题=火针 或者 keyword=中英文扩展(火针) 或者 title=中英文扩展(火针) 或者 abstract=中英文扩展(火针)) 或者 (旧版主题=掀针 或者 keyword=中英文扩展(掀针) 或者 title=中英文扩展(掀针) 或者 abstract=中英文扩展(掀针)) ) ) 或者 ( (旧版主题=皮内针 或者 keyword=中英文扩展(皮内针) 或者 title=中英文扩展(皮内针) 或者 abstract=中英文扩展(皮内针)) 或者 (旧版主题=项针 或者 keyword=中英文扩展(项针) 或者 title=中英文扩展(项针) 或者 abstract=中英文扩展(项针)) ) ) 或者 ( (旧版主题=腕踝针 或者 keyword=中英文扩展(腕踝针) 或者 title=中英文扩展(腕踝针) 或者 abstract=中英文扩展(腕踝针)) 或者 (旧版主题=针 或者 keyword=中英文扩展(针) 或者 title=中英文扩展(针) 或者 abstract=中英文扩展(针)) ) ) (模糊匹配)

WangFang

主题:(帕金森 OR 帕金森病 OR 帕金森综合征 OR 帕金森症 OR 震颤麻痹 OR 颤证) and 主题:(吞咽 OR 吞咽障碍 OR 吞咽困难) and 主题:(针灸 OR 针刺 OR 毫针 OR 电针 OR 火针 OR 皮内针 OR 掀针 OR 腕踝针 OR 耳针 OR 温针 OR 针法 OR 经皮穴位电刺激 OR 穴位按压)

**VIP**

(((((((题名或关键词=帕金森 OR 题名或关键词=帕金森病) OR 题名或关键词=帕金森综合征) OR 题名或关键词=帕金森症) OR 题名或关键词=震颤麻痹) OR 题名或关键词=颤证) AND (((题名或关键词=吞咽 OR 题名或关键词=吞咽障碍) OR 题名或关键词=吞咽困难) OR 题名或关键词=吞咽功能)) AND ((((((((((((题名或关键词=针灸 OR 题名或关键词=针刺) OR 题名或关键词=毫针) OR 题名或关键词=电针) OR 题名或关键词=火针) OR 题名或关键词=皮内针) OR 题名或关键词=掀针) OR 题名或关键词=腕踝针) OR 题名或关键词=耳针) OR 题名或关键词=温针) OR 题名或关键词=针法) OR 题名或关键词=经皮穴位电刺激) OR 题名或关键词=穴位按压))

**CBM**

( "帕金森"[常用字段:智能] OR "帕金森病"[常用字段:智能] OR "帕金森综合征"[常用字段:智能] OR "帕金森症"[常用字段:智能] OR "震颤麻痹"[常用字段:智能] OR "颤证"[常用字段:智能]) AND( "吞咽"[常用字段:智能] OR "吞咽障碍"[常用字段:智能] OR "吞咽困难"[常用字段:智能] OR "吞咽功"[常用字段:智能]) AND( "针灸"[常用字段:智能] OR "针刺"[常用字段:智能] OR "毫针"[常用字段:智能] OR "电针"[常用字段:智能] OR "火针"[常用字段:智能] OR "皮内针"[常用字段:智能] OR "掀针"[常用字段:智能] OR "腕踝针"[常用字段:智能] OR "耳针"[常用字段:智能] OR "温针"[常用字段:智能] OR "针法"[常用字段:智能] OR "经皮穴位电刺激"[常用字段:智能] OR "穴位按压"[常用字段:智能])

# Supplementary Figures and Tables

## Supplementary Figure


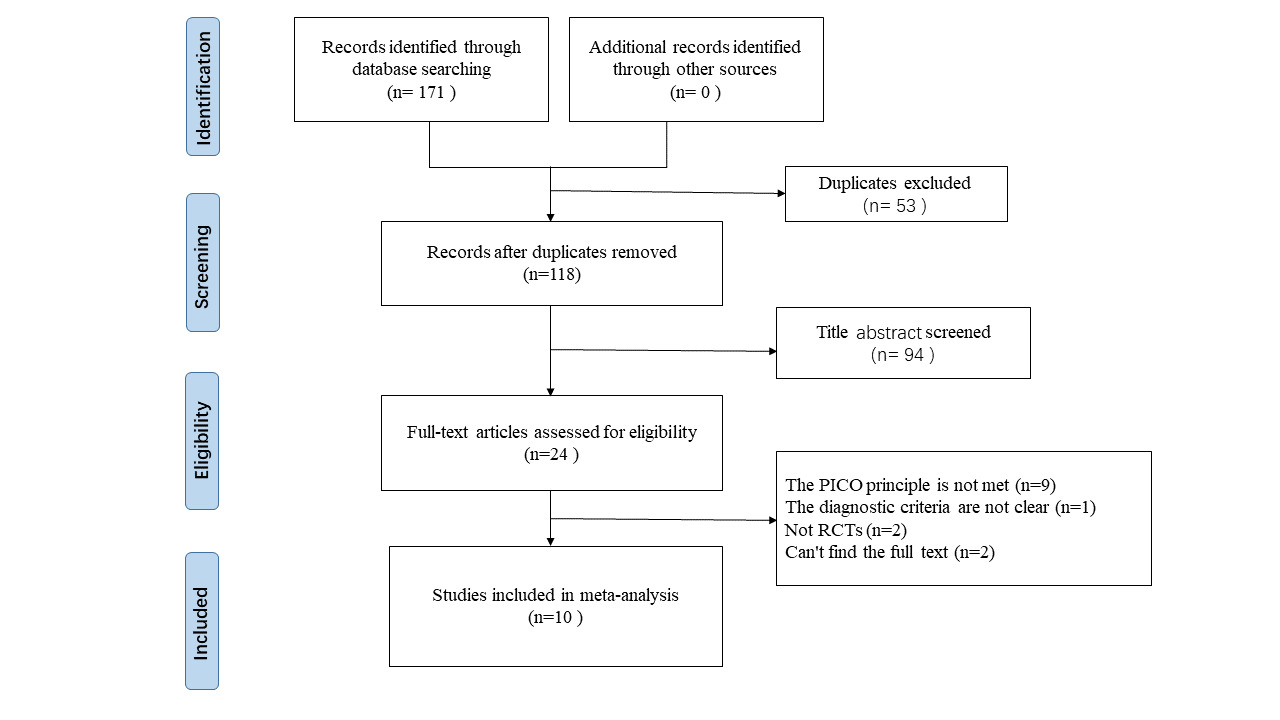


**Supplementary Figure 1.** Flow diagram of searching and articles selection.

**
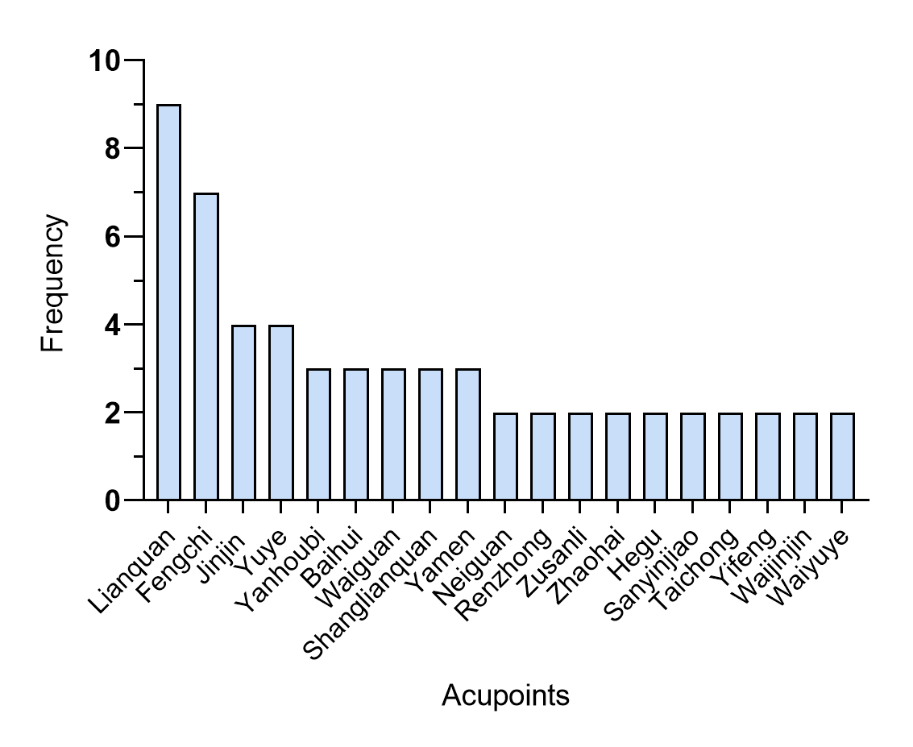
**

**Supplementary Figure 2.** Frequency of commonly used acupoints.


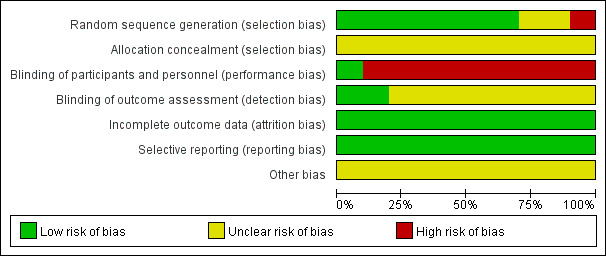


**Supplementary** **Figure** **3**. Risk of bias graph.


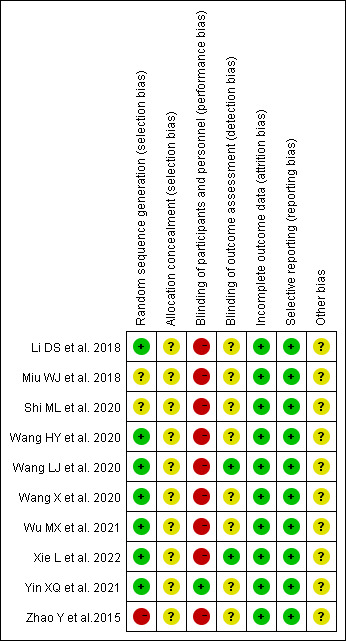


**Supplementary Figure 4.** Risk of bias summary.


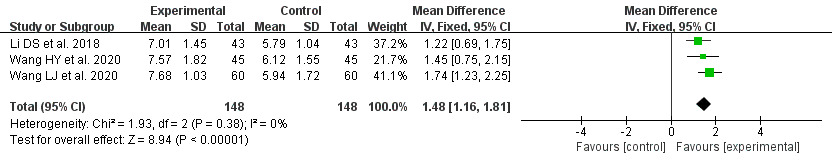


**Supplementary Figure 5.** Forest plot of VFSS scores comparison between acupuncture and control group.


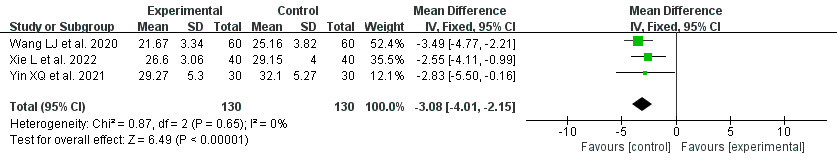


**Supplementary Figure 6.** Forest plot of SSA scores comparison between acupuncture and control group.


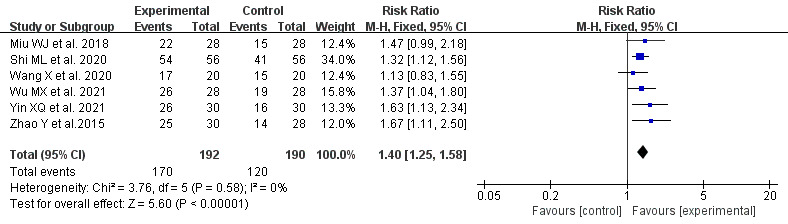


**Supplementary Figure 7.** The forest plot shows a comparison of total eﬃciency rates between the acupuncture and the control group.


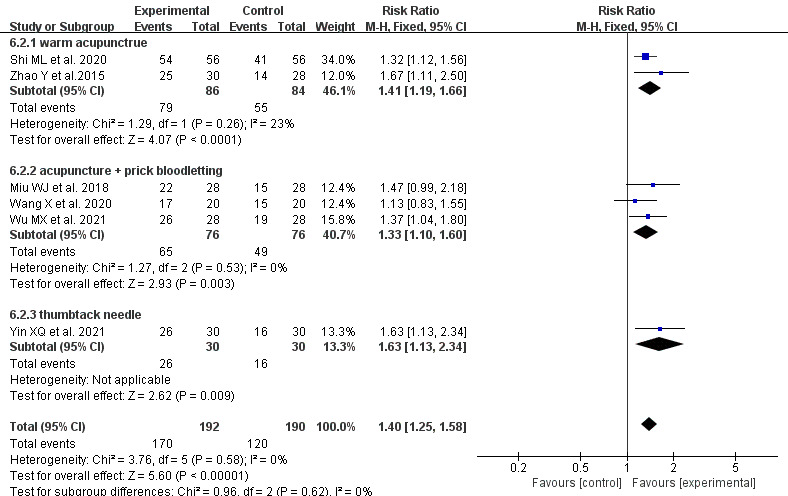


**Supplementary Figure 8.** The forest plot shows a comparison of effectiveness in treating Parkinson's dysphagia between the acupuncture and the control group, based on a subgroup analysis of different acupuncture methods.


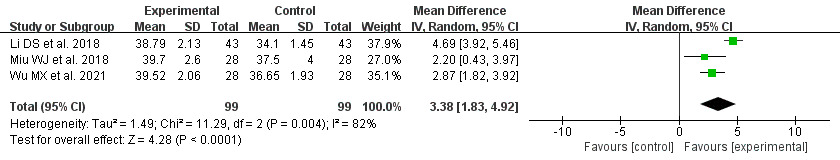


**Supplementary Figure 9.** Forest plot of ALB level in comparison between acupuncture group and control.


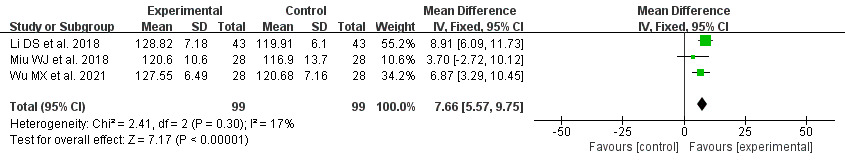


**Supplementary Figure 10.** Forest plot of Hb level in comparison between acupuncture group and control.


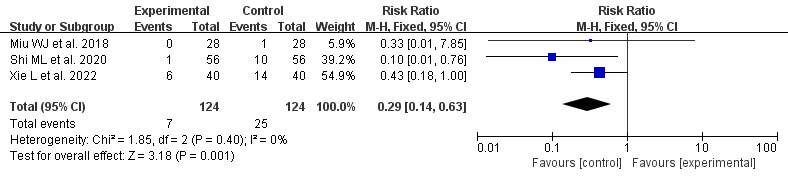


**Supplementary Figure 11.** Forest plot comparing the incidence of pulmonary infection between the acupuncture and the control group.

## Supplementary Figures and Tables

Table 1 Characteristics of included studies.

| Study | Sample Size (T/ C) | Age (mean±SD) | Disease Duration | Invention | | Duration of Treatment | Acupoints | Control | | Outcome | |
| --- | --- | --- | --- | --- | --- | --- | --- | --- | --- | --- | --- |
| Zhao Y, 2015 | 30/28 | C:36-70  T:40-69 | N/A | WA+FT | 30 minutes each day | | YaMen(DU15),LianQuan(RN23),JuQuan(EX-HN10),FengChi(GB20),RenZhong(DU26),NeiGuan(PC6),ZuSanLi(ST36) | | FT | ① |  |
| Li DS, 2018 | 43/43 | C:58.46±4.3 T:59.37±4.89 | C:(6.01±1.25)y T:(5.80±1.43)y | EA+FT | 5 times a week for 30 minutes,4weeeks | | LianQuan(RN23),YiFeng(SJ17),FengChi(GB20),WanGu(GB12),WaiYuYe,WaiJinJin | | FT | ①②④⑤ |  |
| Miu WJ, 2018 | 28/28 | C:67.88±8.53T:67.50±9.70 | C:(5.23±2.14)y T:(5.25±2.67)y | A+P+FT | Once a day, 4weeeks. | | LianQuan(RN23),ShangLianquan,JinJin YuYe(EX-HN12) | | FT | ①④⑤⑥ |  |
| Shi ML, 2020 | 56/56 | C:55.52±1.14T:65.58±1.16 | C:(4.52±0.26)w T:(4.54±0.24)w | WA+FT | 30 minutes each day | | RenZhong(DU26),YaMen(DU15),LianQuan(RN23),NeiGuan(PC6),FengChi(GB20) | | FT | ①⑥ |  |
| Wang X, 2020 | 20/20 | C:52-70  T:50-72 | C:(228±136)d T:(234±140)d | A+P+FT | 30 minutes each day,20-30days | | SheJian,JinJin YuYe(EX-HN12),YanHouBi,BaiHui(DU20),LianQuan(RN23),HeGu(LI4),Quchi(LI11),WaiGuan(SJ5),TaiChong(LR3),ZuSanLi(ST36),SanYinJiao(SP6); point selection by syndrome differentiation | | FT | ① |  |
| Wang HY, 2020 | 45/45 | C:59±10  T:59±10 | C:(5.26±1.02)y T:(5.31±1.08)y | A+P+FT | 5 times a week for 30 minutes,4 weeks | | ShenTing(DU24),BaiHui(DU20),ShangLianQuan,YinTang(DU29),TianZhu(BL10),FengChi(GB20),WaiGuan(SJ5),JinJin YuYe(EX-HN12),ZhaoHai(KI6),LieQue(LU7),YanHouBi | | FT | ①② |  |
| Wu MX, 2021 | 28/28 | C:65±7  T:63±10 | C:(5.4±3.2)y T:(5.2±3.3)y | A+P+FT | 5 times a week for 30 minutes,6 weeks | | LianQuan(RN23),ShangLianQuan,FengChi(GB20),WaiGuan(SJ5),FengFu(DU16),YaMen(DU15),NeDaYing,JinJin YuYe(EX-HN12),YanHouBi | | FT | ①②④⑤ |  |
| Wang LJ, 2020 | 60/60 | C:52.0±11. T:54.0±9.2 | C:(1-2)y T:(1-2)y | A+FT | 6 times a week for 30 minutes,4 weeks | | FengChi(GB20),YiMing(EX-HN13),GongXue,TunYan,LianQuan(RN23),WaiYuYe,WaJjinJin, | | FT | ②③ |  |
| Yin XQ, 2021 | 30/30 | C:65±5.25 T:63.17±5.02 | C:(4.85±5.40)y T:( 4.60±5.65)y | TN+FT | Once every two days for 24 hours each time | | LianQuan(RN23),YiFeng(SJ17),JiaLianQuan,JiaJiXue(C3,C4,C5) | | FT+SM | ①③ |  |
| Xie L, 2022 | 22/24 | C:64.8±5.5 T:65.3±5.4 | C:(6.26±1.62)y T:(6.28±1.50 )y | A+M | 6 times a week for 30 minutes,4 weeeks | | TaiXi(KI3),ZhaoHai(KI6),BaiHui(DU20),GuanYuan(RN4),SanYinJiao(SP6),TaiChong(LR3),HeGu(LI4),FengChi(GB20)XueHai(SP10),LianQuan(RN23),PangLianQuan | | NMES+M | ②③⑥⑦ |  |

Notes: C: Control; T: Treatment; y: year; d: day; w: week; A: Acupuncture; WA: Warming acupuncture; TN: Thumbtack needle; P: Prick bleeding; FT: Functional training; SM: Sham acupuncture; M: Medicine; NMES: Neuromuscular electrical stimulation; N/A: Not applicable. ①: Water swallow test (WST); ②: Videofluoroscopic Swallowing Study (VFSS) scores; ③: Standardized Swallowing Assessment (SSA) scores; ④: Albumin (ALB); ⑤: Hemoglobin (Hb); ⑥: Incidence of pulmonary infection; ⑦adverse events.
